# Establishing an analytic pipeline for genome-wide DNA methylation

**DOI:** 10.1186/s13148-016-0212-7

**Published:** 2016-04-27

**Authors:** Michelle L. Wright, Mikhail G. Dozmorov, Aaron R. Wolen, Colleen Jackson-Cook, Angela R. Starkweather, Debra E. Lyon, Timothy P. York

**Affiliations:** School of Nursing, Yale University, West Haven, CT USA; Department of Biostatistics, Virginia Commonwealth University, Richmond, VA USA; Center for Clinical and Translational Research, Virginia Commonwealth University, Richmond, VA USA; Departments of Pathology and Human and Molecular Genetics, Virginia Commonwealth University, Richmond, VA USA; School of Nursing, University of Connecticut, Storrs, CT USA; College of Nursing, University of Florida, Gainesville, FL USA; Department of Human and Molecular Genetics, Virginia Commonwealth University, Richmond, VA USA

**Keywords:** DNA methylation, Epigenomics, Microarray analysis

## Abstract

The need for research investigating DNA methylation (DNAm) in clinical studies has increased, leading to the evolution of new analytic methods to improve accuracy and reproducibility of the interpretation of results from these studies. The purpose of this article is to provide clinical researchers with a summary of the major data processing steps routinely applied in clinical studies investigating genome-wide DNAm using the Illumina HumanMethylation 450K BeadChip. In most studies, the primary goal of employing DNAm analysis is to identify differential methylation at CpG sites among phenotypic groups. Experimental design considerations are crucial at the onset to minimize bias from factors related to sample processing and avoid confounding experimental variables with non-biological batch effects. Although there are currently no de facto standard methods for analyzing these data, we review the major steps in processing DNAm data recommended by several research studies. We describe several variations available for clinical researchers to process, analyze, and interpret DNAm data. These insights are applicable to most types of genome-wide DNAm array platforms and will be applicable for the next generation of DNAm array technologies (e.g., the 850K array). Selection of the DNAm analytic pipeline followed by investigators should be guided by the research question and supported by recently published methods.

## Background

Epigenetic changes, among which DNA methylation (DNAm) is frequently studied, are increasingly being considered as potential contributors to disease processes or to serve as biomarkers for patients at risk of developing disease [1]. Clinical research conducted to identify biological mediators associated with an increased risk for disease suggest that epigenetic modifications may be involved in virtually any complex disease [2]. In contrast to genetic mutations that arise infrequently in somatic cells and tend to be relatively static, epigenetic modifications are plastic. As a result, these alterations may be amenable to clinical interventions and could lead to the development of new treatments [3]. In recent years, there has been a proliferation of translational clinical research utilizing data produced by high-throughput technologies that are capable of measuring epigenomic modifications across the genome [4]. Generally speaking, high-throughput technologies allow for the simultaneous measurements of thousands of DNA features (e.g., CpG sites) in a more rapid, reproducible, and cost-effective manner than assessing each feature individually. These genome-wide platforms have the advantage of obtaining a snapshot of the cellular state and have revolutionized the genome sciences by facilitating hypothesis-generating studies to complement more direct hypothesis testing. The Illumina Infinium HumanMethylation450 BeadChip (450K) is the most commonly used tool to assess genome-wide DNAm according to the Gene Expression Omnibus Database [5], and the technical details of this technology have been previously published [6]. Best practices for designing whole-genome DNAm experiments have also been published [7–9]. The purpose of this review is to provide an accessible summary of the major data processing steps routinely applied in clinical studies investigating genome-wide DNAm using the 450K array. Although DNAm technology continues to change at a rapid pace, the information in this review will be applicable to the next generation of DNAm array technologies [10].

## Review

### Measurement of genome-wide DNAm

DNAm is a chemical modification to DNA that adds a methyl group to cytosines that are adjacent to guanine nucleotides, referred to as CpG sites, which may result in changes to gene expression without altering the DNA sequence [11]. The relationship between this chemical modification and health conditions has been increasingly studied, and several investigators report associations between the degree of methylation in a genomic region in disease states or in response to environmental exposures [1, 12]. DNAm across the genome can be assayed using either microarrays or specialized whole-genome sequencing (WGS) protocols. In both approaches, DNA samples are first treated with sodium bisulfite, which converts unmethylated cytosines to uracil, while methylated cytosines are unaffected. Microarrays measure DNAm using thousands of oligonucleotide probes that each target a specific genomic location [6]. WGS provides comprehensive coverage of DNAm sites; however, it has a much higher cost and many investigators have elected to use less expensive modern DNAm arrays for initial surveys of DNAm in epidemiological samples [13].

The most frequently used microarrays for measuring DNAm are manufactured by Illumina. The first Illumina methylation microarray, referred to as the 27K array, interrogated 27,578 sites across the genome. For this array, two probes were used for each locus to separately identify methylated and unmethylated CpG sites. The next major methylation platform released by Illumina was the Infinium Human Methylation450 BeadChip microarray, which measures 485,512 CpG sites, the majority of which are localized to regions that potentially could regulate gene expression and therefore are of possible clinical relevance (99 % of the sites are localized to genes that have been well characterized in RefSeq or sites outside of genes that are likely to regulate gene expression, such as promoter regions) [6]. In addition to the dual-probe approach used in the 27K array, which is referred to as a “type I” probe, the 450K array incorporates “type II” probes to expand CpG site coverage. The type II probes use two differentially “colored” (using fluorochromes) channels for each locus, one that is specific for methylated and one for unmethylated CpGs, with these channels being distinguished from one another by a single-base mismatch.

**Fig. 1:**
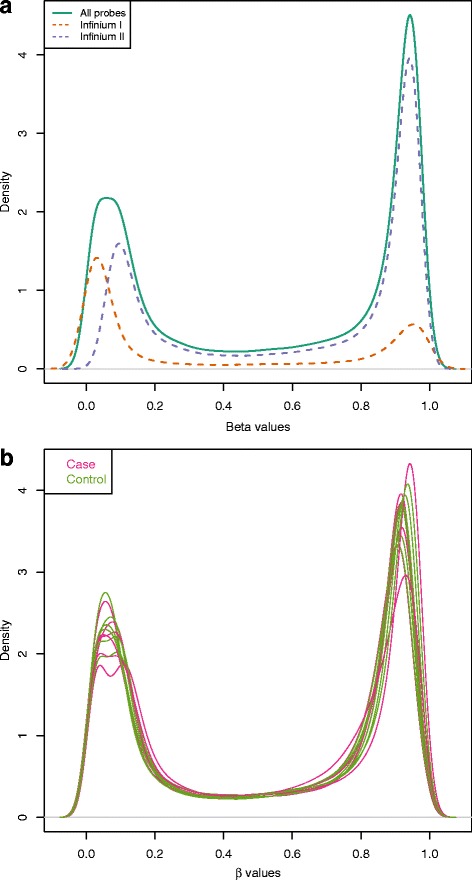
**a** Density of DNAm intensity by probe type. Infinium I and II assays display different *β*-value distributions (0 indicating unmethylated sites, 1 indicating fully methylated sites), which may lead to results that contain an over-representation of type I probes due to the larger variance of type II assays. This figure shows the distribution of *β*-values that were obtained from a single peripheral blood specimen collected for women diagnosed with breast cancer. Differences in between probe types (visualized at the ends of the distributions (type I probes—*red dotted line*; type II probes—*blue dotted line*)) are adjusted using normalization procedures, which attempt to harmonize the differences in distributions between probe types. **b** Density of DNAm intensity by the experimental group. The quality of the data for each specimen can be readily visualized using a density plot, which enables one to compare distributions between, for instance, cases and controls in order to identify particular specimens with deviations in their distribution, the latter of which may serve as an indication that the specimen results are of poor quality

Signal processing for all array-based technologies requires a multi-step analysis protocol to obtain consistent and reproducible results [14, 15]. For example, the 450K array has values for type I probes (135,476 sites), as well as type II probes (350,036 sites), the latter of which measure both methylated and unmethylated CpGs (using two color channels, as noted above) [6]. Technical differences in the probe designs result in less efficiency for detecting variance in DNAm in the type II probes when compared to type I probes, especially at the ends of the probe intensity distribution (Fig. 1a). As a result of the “hybrid” two-assay (type I and type II probes) design of the 450K array, researchers have developed several statistical approaches to normalize variance between the probe types and reduce a potential source of bias in results favoring one probe type over another [16–20]. Signals for both probe types are frequently summarized as a beta value representing a ratio of the average signal for methylated alleles to that of the total of the methylated and unmethylated alleles for biological interpretation [6].

The goal of this methods review is not to describe the technical details of data analysis approaches but rather to outline the major steps/considerations in the experimental design and processing of raw data into interpretable DNAm values. By understanding the analytic pipeline, a clinical researcher will be better able to plan and communicate project goals as well as have appropriate expectations related to the interpretation of results. Software for all methods presented can be obtained through the free and open-source R statistical computing environment [21] and Bioconductor [22] (cran.r-project.org and bioconductor.org). A selection of popular DNAm packages have been reviewed elsewhere [16, 20]. These R and Bioconductor resources have become the de facto software for methods development in this area by an expert community of researchers who continually introduce and upgrade analytic “packages” by taking advantage of R’s highly extensible platform. A summary of the data processing steps described in this review is listed in Table 1.

**Table 1:** Major steps in the 450K array analysis pipeline

Analysis	Rationale
Sample filtering	Experimental samples are compared to control probes present within the array technology to identify samples that fail to adequately detect DNAm. Samples with poor detection may be inaccurate, due to poor sample quality, and thus might be considered for exclusion from the dataset.
Probe filtering	Raw data must past initial quality and data screening. Probes failing to meet preset detection values and/or failed probes are removed from analysis because they are unreliable (see text). For example, some probes may cross-hybridize or overlap with SNPs, which could confound results. Study aims should be considered when determining which probes to remove.
Within-array normalization	This step removes “background” noise and corrects for technical dye-based (red/green), intensity, and probe type (I/II) differences within the array technology.
Batch effects	The step assesses and accounts for variation that is not caused by biological differences but by external variation (e.g., samples are processed on different days or at different facilities).
Cell composition	Whole blood contains multiple cell types with potentially different DNAm profiles. As different samples may contain varying proportions of cell types, statistical methods have been developed to estimate and correct for this cellular heterogeneity.
Differential DNAm positions and regions	Currently, many analytic pipelines assess for DNAm differences in both specific positions and broader regions. DNAm positions interrogated on the array are not evenly distributed, and both differentially methylated positions and regions may yield clinically meaningful results.
Biological and clinical interpretation	Various approaches may be necessary for accurate interpretation of differential methylation between groups. Tools for functional and regulatory enrichment analyses are available. Manual exploration of the literature and validation in a second cohort or by another method (e.g., bisulfite sequencing) remains as viable options for interpretation.

## Overview of DNAm array analysis pipeline

### DNAm analysis software

Illumina provides a default application called GenomeStudio to analyze their various microarray technologies, which includes a DNAm module. Being graphical user interface (GUI)-based (i.e., point and click), GenomeStudio is relatively easy to use and provides convenient methods for generating common visualizations. However, its data processing capabilities are limited, and it lacks support for the more cutting-edge and effective analysis methods.

We recommend analyzing DNAm data using the free and open-source R programming language [21]. R was designed specifically to facilitate statistical computing and provide a highly extensible platform upon which novel methods could be easily built and distributed as “R packages.” R has become the data analysis tool of choice in a wide range fields and is the de facto tool in genomics research largely due to the Bioconductor project, which provides hundreds of R packages for analyzing genomic data [22]. All methods presented here are freely available as R packages published by Bioconductor [16, 20].

A significant benefit to using R is the enhanced reproducibility it provides over GUI-based applications. As in any programming language, analyses are performed by writing and executing scripts. These scripts are effectively self-documenting, providing a step-by-step record of what was done. When performing analyses as multifaceted as those described here, where each step can have significant downstream consequences, methodological transparency is critical for results to be reproducible. R also provides methods for implementing analyses within a “dynamic document” where analytical code and expository text are combined within a single document, which enhances reproducibility and facilitates communicating results [23].

### Sample filtering

The 450K array contains several different types of control probes to assist in determining the quality of the array experiment. For example, these metrics include probes to assess bisulfite conversion efficiency and background fluorescence levels and have been designed to help the investigator identify “wet lab” experimental steps that might have been completed sub-optimally [15]. In general, control probe intensity values that fall outside of the clustering of values for other samples could indicate a compromised/failed sample. We have found that the method proposed by the R minfi [24] package provides a reasonable and straightforward assessment of sample quality that oftentimes corresponds to the aforementioned deviation in control probes. In this method, samples of good quality tend to group together based upon clustering the log median intensities of the raw methylated values against those of the unmethylated values for each array, and poorer quality arrays will tend to deviate towards lower median values in both dimensions [24]. Additionally, a comparison of beta value density plots (Fig. 1b) from an experiment can identify poor performing arrays based on a large deviation from the rest of the samples. However, care should be taken to decide whether homogeneity of beta value distributions should be assessed within experimental classes insofar as the observed differences may have biological relevance and reflect expected deviations that are not related to technical artifact (i.e., cancer versus control samples).

### Probe filtering

Probe filtering provides the opportunity to eliminate specific CpG probes that do not meet quality control standards or fulfill study objectives. Standard filtering approaches include eliminating probes with (1) intensity levels at or near background intensity; (2) poorly represented CpG sites; and (3) variable target sequences. The 450K array includes over 600 negative control probes, which can be used to estimate background intensity levels. Probes with intensity values that are not statistically greater than this background value (frequently, a *P* value >0.01) could be unreliable and considered as failed probes. One approach for probe-specific filtering is to remove probes that fail to measure DNAm in a specified proportion of the total samples (e.g., 10–25 %). Each CpG locus on the 450K array is targeted by a specific probe sequence affixed to a bead, with the median number of beads per locus being 14, which are randomly distributed across the array. A filtering criterion can be used to identify probes that have failed to hybridize if a minimum of three beads are not detected on the array [16]. Probes located where the DNA sequence contains known single nucleotide polymorphisms (SNPs) are also often excluded from statistical analysis because these SNPs can disrupt probe binding at the site and artificially lower intensity signals [6, 17, 25]. If SNP sites are not removed from the analysis, one could encounter difficulties interpreting differences in measured DNAm values at these sites since these differences could reflect a variation in DNA sequence (e.g., C/A SNP variation) or true DNAm differences. If sequence variation can be validated in the study, further analysis can be completed to evaluate the relative contribution of DNAm differences by variant type (i.e., SNP versus DNAm).

The research objectives and experimental design of a study should dictate the appropriate filtering strategy to be used. For instance, the removal of Y chromosome probes may be indicated for investigations restricted to a study of only females. Yet, for most applications, a conservative approach is usually adopted by omitting probes with SNPs that fall at the CpG target or at the single-base extension sites. In addition to SNPs, CpG probes located near short insertions and deletions, and probes that map to multiple locations on the genome, may also produce results that are difficult to interpret and should be removed if there are no plans to validate these findings within the present study [17, 26].

### Within-array normalization

The observed probe intensity level can be broken down into component parts consisting of measurement of the true intensity level (i.e., “signal”) and intensity measurement due to technical artifacts (i.e., “noise”). Increasing the signal to noise ratio by reducing variation due to extraneous sources is an essential step in the processing of all microarray platforms. For the 450K design, the primary adjustments required pertain to three technical variations: (1) non-specific background fluorescence; (2) red/green dye bias; and (3) rescaling for probe type (I/II) differences [16]. More advanced model-based background correction methods take advantage of the 450K array technology to measure the intensity level of type I probes outside of their specified color band (*N* = 135,501 probes) and have been shown to be superior to subtractive methods that rely exclusively on the negative probes (*N* = 42) [15, 27]. The normal-exponential convolution using out-of-band probes (noob) method has been shown to provide a comprehensive background correction and dye normalization and sufficiently scale type I and II probes to make the beta values for these probe comparable [15]. Other artifacts to consider include spatial heterogeneity across individual arrays and the BeadChip slide [27] and non-specific binding due to cross-hybridization [28]. While there is no consensus framework for handling this technical variation, several investigators have compared potential solutions for statistical adjustment and assessment [29, 30].

The 450K array can be viewed essentially as two arrays each with a different probe chemistry (i.e., type I and II as described above). As noted earlier, Fig. 1a displays the characteristic bimodal distribution of beta values with modes near the fully methylated (*β* = 1) and unmethylated (*β* = 0) extremes. The shift in peaks corresponding to probe type is primarily due to assay sensitivity. These differences can have downstream effects on analysis since the type II probes show a smaller range of beta values and exhibit larger variance between repeated measures compared with the type I probes [20, 25]. This, along with the fact that placement of probes differ in functional regions, could lead to a biased detection of differentially methylated regions enriched for type I probes. A large amount of attention has been focused on the rescaling of probe distributions to make them comparable and several options exist [31–35]. One approach that has been used to address this problem is to apply quantile normalization, such as the version adapted for the 450K array in the minfi R package [24, 32]. The major assumption underlying this method is that only modest changes are expected between experimental classes. An assumption free normalization procedure, such as Funnorm [34], is an alternative method that can be used to address this issue and has strength for evaluating specimens where a global DNAm shift is expected, such as in the case of cancer to normal sample comparison studies. Developers frequently publish detailed comparisons of methods (new versus existing) with analysis of downstream effects, and several reviews can be found within the following references [16, 24, 28, 30–36].

### Batch effect analysis and correction

Data produced by microarray technologies, like the 450K array, are susceptible to batch-to-batch variation [37–39]. Batch effects have been identified as a major confounding factor in genome-wide DNAm studies, especially when batch group is correlated with the outcome of interest, such as change over time [37]. The term “batch” refers to a grouping of samples that undergo an experimental processing step in tandem, potentially introducing DNAm differences that reflect differences between batches and not in experimental factors of interest. This pervasive batch influence is almost unavoidable for samples collected over a large period of time [37]. In the extreme case, if all control samples are run in the same batch and the intervention group run in a separate batch, significant differences in results between groups could merely be due to the average effect of extraneous factors unique to each batch [40]. A batch effect can conceivably be introduced at a number of points during specimen processing and sample analysis.

The most common type of batch effect is observed when experimental procedures necessitate the processing of samples in separate groups or on different days. This and other similar sources of technical artifact can be minimized by carefully randomizing experimental groups across array processing steps. The effects of known batches can be, if necessary, controlled for in downstream analyses through appropriate statistical modeling insofar as the experimental group and batch membership are not completely confounded. Yet, it is still possible that an unobserved batch structure can exist. Several methods are available to correct for unobserved batch effects, which take advantage of the large number of measurements obtained by high-throughput technologies to identify and test for the presence of unobserved correlation structure [39, 41].

### Cell composition correction

DNAm can vary by cell type and can confound analysis when pooled samples are being investigated. For example, peripheral blood samples, which are comprised of several different cell types (albeit with some of the types in small proportions (less than 5 to 10 % of cells)), are frequently used for identifying DNAm differences in clinical populations [42]. As DNAm signatures are different among cell types, a sample with abnormal cell-type proportions (e.g., an abnormally high eosinophil count caused by an allergy) may result in the identification of significant DNAm differences due merely to differences in cell-type proportions present in the specimen, the latter of which may not be related to the health condition being evaluated in the investigation. Statistical corrections can be performed to estimate heterogeneity of cell types found in peripheral blood based on DNAm previously identified in purified blood cell lines [42]. Research evaluating DNAm in other mixed cell tissues (e.g., placenta), where a specific cell type has not been isolated prior to analysis (e.g., syncytiotrophoblasts), can be adjusted for cell-type heterogeneity using recent innovations in reference-free approaches such as EWASher [43] and RefFreeEWAS [44].

### Calculation of differentially methylated positions and regions

**Fig. 2:**
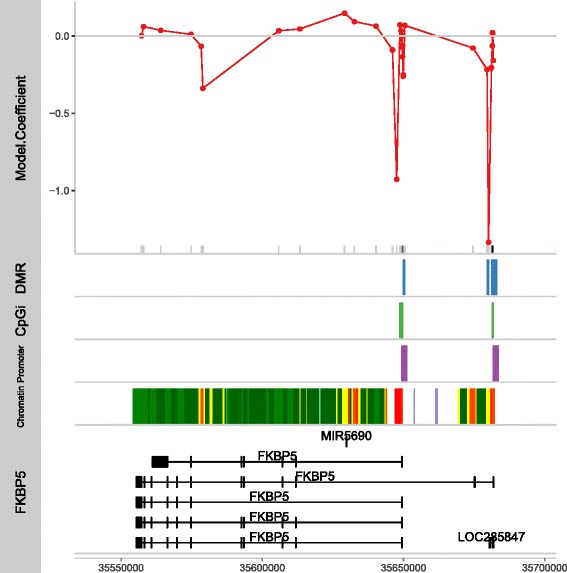
Visualization of DMR and DMP results overlapping genomic annotations. This example figure, which was created from 450K data for peripheral blood specimens that were collected from women diagnosed with breast cancer, demonstrates how both experimental results and predicted functional elements can be viewed as individual tracks along a set of genomic coordinates (*x*-axis) specified along a gene (e.g., the FKBP5 gene (*bottom track*)). The *top track* displays the statistical model coefficient from a univariate test to identify individual DMPs, and the *plot rug* (along the *x*-axis) indicates significant (*black tick*) findings. The identified DMRs (*second track*) correspond to clusters of DMP results with similar coefficient values and in this example overlap CpG islands (*third track*) and predicted promoter regions (*fourth track*). These regions also correspond to other publically curated annotations that, for instance, can indicate enrichment for different chromatin states (*fifth track*)

The statistical analysis of high-throughput DNAm technology presents challenges that are similar to other high-throughput technologies (e.g., GWAS and gene expression microarrays), as well as those unique to the 450K array technology. The results of numerous DNAm studies have reported a strong correlation of DNAm levels at neighboring CpG sites that decrease as pairwise distances increase [37, 45, 46]. This spatial correlation can be due to both coordinated DNAm change and measurement errors. Recently, investigators have taken advantage of this structure by developing analytic strategies to discover differentially methylated regions (DMR) composed of multiple signals across individual CpG positions (differentially methylated positions (DMP)) [37, 46–50]. It has been shown that the identification of regional differences across several probes provides more robust findings and is more likely to be replicated than individual CpG differences [46, 51]. Yet, the identification of DMRs using the 450K array remains a challenge due to the sparse and non-uniform placement of probes on the array (see Fig. 2). For instance, the “bump hunting” procedure of Jaffe and colleagues [37], designed for the high-density CHARM platform, would only be applicable to approximately 20 % of the 450K array [46]. Additionally, since most DMR methods estimate significance using permutation-based methods, these approaches are not easily extended to more complex experimental designs and custom algorithms are required [46]. It is recommended that both DMR and DMP approaches be run in tandem since, as mentioned previously, probes are not evenly spaced across the genome and a significant proportion of CpG sites are not positioned within even 1 kb of a neighboring site.

Another consideration when performing statistical tests includes the transformation of beta values to *M*-values (logit beta values) to promote normality and reduce the heteroscedasticity at extreme beta values [52]. Additional innovations, such as independent filtering, can be used to improve statistical power and reduce the multiple testing burden in high-dimensional data [53, 54]. Although region-based analyses (e.g., DMR) can be considered a data reduction technique, it is unlikely that it will eliminate the need for a multiple-test correction to reduce the false discovery rate [8, 20, 55].

### Biological and clinical interpretation

The interpretation, or recognition of biological relevance of differential DNAm, is arguably the most important step concluding the well-planned bioinformatics analysis of 450K data [56]. This step may be used to reveal potential mechanistic underpinnings of clinically relevant information (e.g., disease processes). There are several interpretation-oriented approaches aimed at understanding the biological and clinical significance of DNAm data. These include comparing results to other publically available datasets, using functional enrichment analysis to determine functions, comparing results to canonical pathways enriched in genes associated with DMRs, and investigating the regulatory context (e.g., histone modification signatures and transcription factor binding sites) of the loci enriched in DMRs.

Comparing the results of DNAm analysis to other publically available datasets stems from a commonly accepted view that genetic similarity correlates with phenotype similarity [57, 58]. This approach has been particularly well developed in gene-centric studies to predict functions of unannotated genes based on co-expression patterns [59–61]. Less attention has been paid to comparing patterns of DNAm, although numerous dedicated databases of DNAm experiments exist [62–64], including the well-known Gene Expression Omnibus (GEO, [5]). The DNAm pattern matching approach for interpretation of newly acquired DNAm data remains confined to individual bioinformatics cores but is expected to gain wider popularity as the pre-processing steps for DNAm data obtained with 450K technology will be better standardized.

The functional enrichment analysis is a well-established method to reveal biological roles of differentially expressed genes [65, 66]. Owning to the fact that the 450K array is designed to interrogate the methylation status of CpG sites in proximity to genes, the functional enrichment analysis is scalable to the interpretation of DMRs through mapping them to the nearby genes. Thus, the functional enrichment analysis generally is performed on DMRs localized within genes, as well as nearby genes. The mapping step is usually achieved by associating the probe ID numbers of each differentially methylated CpG site with gene names using a manufacturer-provided mapping (“manifest”) file. If a CpG site maps to several nearby genes, one may elect to use all these genes. After selecting genes mapped to DMRs, the functional enrichment analysis can be performed using standard tools, such as DAVID [67], ToppGene suite [68], or GSEA [69]. The functional enrichment analysis of genes mapped to DMRs is a way of obtaining biologically meaningful insights into molecular mechanisms, biological processes, and canonical pathways potentially affected by DNAm differences.

In interpreting DMR data, it is important to note that a gene-centric analysis of DMRs is an approximation in that it operates under the hypothesis that DMRs affect nearby genes. However, if a CpG site maps to multiple nearby genes, it may be difficult to know which gene is truly regulated by the methylation differences at this CpG site. Given recent availability of functional/regulatory genome annotation data provided by the ENCODE [70] and Roadmap Epigenomics [71] projects, an alternative and/or complimentary approach is to evaluate the regulatory context of the DMRs. This approach, termed “regulatory enrichment analysis,” is similar to the gene-centric functional enrichment analysis in that it evaluates co-localization enrichment of DMRs in different types of regulatory datasets, e.g., gene promoter region, chromatin state [72] (Fig. 2). Given the large volumes of genome annotation data, the regulatory enrichment analysis methods are less well developed than functional enrichment analysis methods. However, tools like GenomeRunner [73], Enrichr [74], and GoShifter [75] have been successfully applied to the interpretation of DMRs identified with 450K technology in studies of autoimmunity [76, 77] and aging [78, 79]. These examples illustrate a variety of methods available for the interpretation of the biological meaning of differentially methylated regions associated with clinical phenotypes.

### Additional considerations

Clinical researchers should maintain realistic expectations for the interpretation of results based on the initial research question, characteristics of the study population under investigation, and the tissue/cells being measured [80]. As described in this review, DNAm variability can be composed of both biological and technical sources and, despite careful experimental design, cannot all be fully accounted for without additional experimentation that may be outside the scope of the current project. For example, the 450K microarray technology cannot, at present, distinguish between hydroxymethylation and DNAm, unless samples undergo a different processing step prior to DNAm quantification [81, 82]. Nor is the microarray technology sensitive enough to detect single-cell DNAm differences and may have limited interpretability for imprinted regions without further laboratory validation of DNAm at specific loci. For example, known regions of allelic imprinting by parent of origin tend to have monomorphic distribution of beta values (i.e., beta value of 0.5) [33] and require interrogation via other methods if determining allele-specific methylation is the project goal. Lastly, the 450K microarray technology has been show to be reproducible across other platforms (e.g., *r* = 0.88 with pyrosequencing [6, 83]), although confidence in research findings can be increased by validating findings using an alternate approach and/or by evaluating plausible downstream products such as concomitant changes in gene expression [8].

## Conclusions

Advances in research methods utilizing new technologies that produce large amounts of data, like the 450K array, are increasingly being used to improve our understanding of various disease processes. Clinical researchers are uniquely poised to accelerate the translational arm of research by using these types of technologies to improve our understanding of disease risk, better estimate disease prognosis, and make more personalized therapeutic decisions. However, the processing and statistical analyses of these data are complex. To ensure accurate translation and clinical applications, researchers must be aware of how these data are processed and analyzed. The analytic pipeline is a foundational process to ensure data quality, reliability, and reproducibility of reported findings. Streamlined approaches are available that automate a number of steps described in this review and can serve as a starting point for less experienced users [16, 56, 84]. This review provides a summation of the essential analytic steps that clinical researchers will need to consider when planning and reporting findings from DNAm studies using the 450K array and can serve as a “prequel” to more in-depth, helpful reviews on specific methods that are available (e.g., [16, 20, 36]).

## Abbreviations

450K: Illumina Infinium HumanMethylation450 BeadChip
DMP: differentially methylated positions
DMR: differentially methylated regions
DNAm: DNA methylation
WGS: whole-genome sequencing
